# First-line nivolumab plus ipilimumab in metastatic non-small cell lung cancer: 5-year outcomes in Japanese patients from CheckMate 227 Part 1

**DOI:** 10.1007/s10147-023-02390-2

**Published:** 2023-08-07

**Authors:** Makoto Nishio, Yuichiro Ohe, Satoshi Ikeda, Toshihide Yokoyama, Hidetoshi Hayashi, Tatsuro Fukuhara, Yuki Sato, Hiroshi Tanaka, Katsuyuki Hotta, Shunichi Sugawara, Haruko Daga, Isamu Okamoto, Kazuo Kasahara, Tateaki Naito, Li Li, Ravi G. Gupta, Judith Bushong, Hideaki Mizutani

**Affiliations:** 1 Cancer Institute Hospital, Japanese Foundation for Cancer Research, 3-8-31, Ariake, Koto, Tokyo, 135-8550 Japan; 2 National Cancer Center Hospital, 5-1-1 Tsukiji, Chuo-ku, Tokyo, 104-0045 Japan; 3 Kanagawa Cardiovascular and Respiratory Center, 6-16-1 Tomiokahigashi, Kanazawa-ku, Yokohama-shi, Kanagawa, 236-0051 Japan; 4 Kurashiki Central Hospital, 1-1-1 Miwa, Kurashiki, Okayama 710-8602 Japan; 5 Kindai University Hospital, 3-4-1 Kowakae, Higashiosaka, Osaka 577-8502 Japan; 6 Miyagi Cancer Center, 47-1 Nodayama, Shiote, Medeshima, Natori, Miyagi 981-1293 Japan; 7 Department of Respiratory Medicine, Kobe City Medical Center General Hospital, 2-1-1 Minatojima-Minamimachi, Chuo-ku, Kobe, 650-0047 Japan; 8 Niigata Cancer Center Hospital, 2-15-3 Kawagishi-cho, Chuo-ku, Niigata, 951-8566 Japan; 9 Okayama University Hospital, 2-5-1 Shikata-cho, Kita-ku, Okayama, 700-8558 Japan; 10 Sendai Kousei Hospital, 4-15 Hirose-machi, Aoba-ku, Sendai, Miyagi 980-0873 Japan; 11 Osaka City General Hospital, 2-13-22 Miyakojima Hondori, Miyakojima Ward, Osaka, 534-0021 Japan; 12 Kyushu University Hospital, 3-1-1 Maidashi, Higashi-ku, Fukuoka, 812-8582 Japan; 13 Kanazawa University Hospital, 13-1 Takaramachi, Kanazawa, Ishikawa 920-8641 Japan; 14 Shizuoka Cancer Center, 1007 Shimonagakubo, Nagaizumi-Cho, Sunto-Gun, Shizuoka 411-8777 Japan; 15 Bristol Myers Squibb, Princeton, NJ USA; 16 Saitama Cancer Center, 780 Oaza Komuro, Ina Machi, Kita-Adachi-gun, Saitama, 362-0806 Japan

**Keywords:** Nivolumab, Ipilimumab, First-line immunotherapy, Japan, 5-year outcomes, Non-small cell lung cancer

## Abstract

**Background:**

In CheckMate 227 Part 1 (NCT02477826), first-line nivolumab plus ipilimumab demonstrated long-term durable overall survival (OS) benefit versus chemotherapy in patients with metastatic non-small cell lung cancer (NSCLC), regardless of tumor programmed death ligand 1 (PD-L1) expression. We report results in Japanese patients with ≥ 5-year follow-up.

**Methods:**

Adults with stage IV/recurrent NSCLC without *EGFR/ALK* aberrations were randomized 1:1:1 to nivolumab plus ipilimumab, nivolumab alone, or chemotherapy (patients with tumor PD-L1 ≥ 1%), or nivolumab plus ipilimumab, nivolumab plus chemotherapy, or chemotherapy (patients with tumor PD-L1 < 1%). Five-year efficacy and safety were assessed in Japanese patients.

**Results:**

At 62.1 months’ minimum follow-up, 143 Japanese patients with PD-L1 ≥ 1% or < 1% were randomized to nivolumab plus ipilimumab (*n* = 66) or chemotherapy (*n* = 77). Five-year OS rates were 46% with nivolumab plus ipilimumab versus 34% with chemotherapy (PD-L1 ≥ 1%) and 36% versus 19% (PD-L1 < 1%). Median duration of response was 59.1 versus 7.1 months (PD-L1 ≥ 1%) and 17.3 versus 3.0 months (PD-L1 < 1%). Among 5-year survivors treated with nivolumab plus ipilimumab (PD-L1 ≥ 1% and < 1%; *n* = 27), 59% (95% CI, 39%–75%) were off treatment for ≥ 3 years without receiving subsequent therapy. No new safety signals were observed.

**Conclusions:**

At 5-year follow-up, nivolumab plus ipilimumab continued to show long-term durable clinical benefit versus chemotherapy, regardless of tumor PD-L1 expression. Consistent with findings for the global population, these data support the use of nivolumab plus ipilimumab as first-line treatment in Japanese patients with metastatic NSCLC.

**Supplementary Information:**

The online version contains supplementary material available at 10.1007/s10147-023-02390-2.

## Introduction

Programmed death 1 (PD-1) and programmed death ligand 1 (PD-L1) inhibitor-based immunotherapy in the first-line setting has significantly improved survival outcomes versus chemotherapy alone for patients with metastatic non-small cell lung cancer (NSCLC) [1–4].

Immune checkpoint inhibitors, nivolumab, a fully human PD-1 antibody, and ipilimumab, a fully human cytotoxic T-lymphocyte antigen 4 antibody, have distinct but complementary mechanisms of action [5]. This dual immunotherapy combination has shown durable survival benefit versus chemotherapy in global clinical trials, in metastatic NSCLC and several other advanced solid tumors [6–8]. CheckMate 227 is a multi-part, randomized, open-label, phase 3 trial evaluating first-line nivolumab-based regimens versus chemotherapy in patients with metastatic NSCLC [1, 9, 10]. In Part 1 of the trial, first-line nivolumab plus ipilimumab showed overall survival (OS) benefit versus chemotherapy in patients with metastatic NSCLC with tumor PD-L1 expression ≥ 1% (independent co-primary endpoint) or < 1% (prespecified descriptive analysis) [1]. At the 5-year follow-up, nivolumab plus ipilimumab continued to provide long-term, durable clinical benefit regardless of tumor PD-L1 expression versus chemotherapy, with no new safety concerns; the majority of 5-year survivors in the nivolumab plus ipilimumab arm did not initiate any subsequent systemic anticancer treatment for ≥ 3 years [11]. Nivolumab plus ipilimumab is approved in the United States as first-line treatment for adults with metastatic NSCLC with no *EGFR/ALK* aberrations and tumor PD-L1 expression ≥ 1%, and in Japan as first-line treatment regardless of tumor PD-L1 expression [12–14].

Differences in treatment outcomes between Asian and non-Asian patients with NSCLC have been reported [15–18]. These may be due to epidemiological and demographical variability and differences in the prevalence of activating driver mutations between populations, such as a high prevalence of *EGFR* mutations in Asian patients with advanced lung adenocarcinoma [16, 19, 20]. As Japan has a well-resourced healthcare system, higher rates of subsequent therapy and better patient management may also impact long-term treatment outcomes [16, 21].

It is therefore important to assess clinical outcomes in Japanese patients to better inform physicians of treatment options. Survival benefit with nivolumab plus ipilimumab across several tumor types has been reported in Asian populations, including Japanese patients [22–24]. At the 3-year follow-up of CheckMate 227, nivolumab plus ipilimumab provided durable long-term efficacy benefits versus chemotherapy, regardless of tumor PD-L1 expression, in the Asian subpopulation, including Japanese patients with metastatic NSCLC [21]. However, long-term clinical data on first-line immunotherapy combinations in Japanese patients remain a high unmet need. Here, we present efficacy and safety results of nivolumab plus ipilimumab versus chemotherapy in Japanese patients from CheckMate 227 Part 1, with a minimum follow-up of 5 years, the longest reported phase 3 outcomes with dual immunotherapy for NSCLC in this subpopulation.

## Patients and methods

### Patients and treatment

Eligibility criteria for CheckMate 227 (NCT02477826) were described previously [1, 9]. Briefly, adults with histologically confirmed squamous or non-squamous stage IV/recurrent NSCLC, with an Eastern Cooperative Oncology Group (ECOG) performance status of 0–1, without prior systemic anticancer therapy for advanced/metastatic disease were enrolled. Patients with sensitizing *EGFR* mutations or known *ALK* alterations, untreated or symptomatic central nervous system metastases, or autoimmune disease were excluded. Patients (*n* = 143) for this subanalysis were enrolled from 32 centers in Japan [21].

Patients with tumor PD-L1 expression ≥ 1% were randomized 1:1:1 to receive nivolumab (3 mg/kg every 2 weeks [Q2W]) plus ipilimumab (1 mg/kg every 6 weeks), nivolumab monotherapy (240 mg Q2W), or platinum-doublet chemotherapy (every 3 weeks [Q3W] for ≤ 4 cycles). Patients with tumor PD-L1 expression < 1% were randomized 1:1:1 to receive nivolumab plus ipilimumab, nivolumab (360 mg Q3W) plus platinum-doublet chemotherapy (Q3W for ≤ 4 cycles), or platinum-doublet chemotherapy (Q3W for ≤ 4 cycles). Treatment continued until disease progression, unacceptable toxicity, or for ≤ 2 years for immunotherapy.

### Endpoints and assessments

The independent co-primary endpoint of OS in patients with tumor PD-L1 expression ≥ 1% and secondary endpoints were previously reported [1]. The efficacy assessments in this 5-year exploratory subanalysis in Japanese patients included OS, progression-free survival (PFS), objective response rate (ORR), and duration of response (DOR) assessed by blinded independent central review (BICR) based on Response Evaluation Criteria in Solid Tumors (RECIST version 1.1) in the tumor PD-L1 ≥ 1%, tumor PD-L1 < 1%, and the combined PD-L1 ≥ 1% and < 1% populations. OS by histology and PFS after the next line of therapy (PFS2) were also assessed by tumor PD-L1 expression. Tumor PD-L1 expression was determined as described previously [1, 25].

Safety and tolerability, including treatment-related adverse events (TRAEs) and immune-mediated adverse events (IMAEs) were assessed in all treated Japanese patients. AEs were graded according to the National Cancer Institute Common Terminology Criteria for Adverse Events, version 4.0. Additional details on AEs are included in the supplementary methods (online only).

Post hoc analyses in Japanese patients included efficacy outcomes (PFS, ORR, and DOR) evaluated in the combined PD-L1 ≥ 1% and < 1% population of patients alive at 5 years, efficacy outcomes (OS, PFS, ORR, and DOR) in the combined PD-L1 ≥ 1% and < 1% population of patients who discontinued study treatment due to TRAEs, and treatment-free interval (TFI; the time from last study dose to start of subsequent systemic therapy or death, whichever occurred first) measured in patients who discontinued study therapy (for any reason), in 5-year survivors, and in patients who discontinued study treatment due to TRAEs.

### Statistical analyses

Efficacy and safety analyses of nivolumab plus ipilimumab versus chemotherapy in the randomized and treated populations, respectively, of Japanese patients were exploratory and summarized using descriptive statistics. Time-to-event analyses for OS, PFS, DOR, and TFI were performed using the Kaplan–Meier method. Hazard ratios (HRs) with associated two-sided confidence intervals (CIs) were calculated using a stratified Cox proportional hazards model, with tumor histology as the strata and treatment group as a single covariate. An unstratified model was used to estimate HRs between treatment arms in patient subgroups. The Clopper-Pearson method was used to calculate 95% exact two-sided CIs for ORRs.

## Results

### Patients

At current database lock (February 15, 2022), the minimum follow-up for OS was 62.1 months and median follow-up was 67.5 months for Japanese patients. In total, 143 Japanese patients with tumor PD-L1 expression ≥ 1% or < 1% from CheckMate 227 Part 1 were randomized to nivolumab plus ipilimumab (*n* = 66) or chemotherapy (*n* = 77) (Supplementary Fig. 1). Baseline characteristics were generally balanced across treatment arms (Table 1).

**Table 1:** Baseline characteristics of Japanese patients by tumor PD-L1 expression

	PD-L1 ≥ 1%		PD-L1 < 1%		PD-L1 ≥ 1% and < 1%	
	Nivolumab plus ipilimumab (*n* = 41)	Chemotherapy (*n* = 48)	Nivolumab plus ipilimumab (*n* = 25)	Chemotherapy (*n* = 29)	Nivolumab plus ipilimumab (*n* = 66)	Chemotherapy (*n* = 77)
Age, median (range), years	66 (43–77)	66 (41–78)	64 (42–81)	66 (30–78)	66 (42–81)	66 (30–78)
Age category, years						
< 65	17 (41)	20 (42)	13 (52)	13 (45)	30 (45)	33 (43)
≥ 65 to < 75	23 (56)	24 (50)	9 (36)	14 (48)	32 (48)	38 (49)
≥ 75	1 (2)	4 (8)	3 (12)	2 (7)	4 (6)	6 (8)
Female	9 (22)	11 (23)	3 (12)	6 (21)	12 (18)	17 (22)
ECOG PS						
0	16 (39)	23 (48)	10 (40)	12 (41)	26 (39)	35 (45)
1	25 (61)	25 (52)	15 (60)	17 (59)	40 (61)	42 (55)
Smoking status^a^						
Current/former smoker	38 (93)	41 (85)	20 (80)	25 (86)	58 (88)	66 (86)
Never-smoker	3 (7)	7 (15)	5 (20)	4 (14)	8 (12)	11 (14)
Histology						
Squamous	13 (32)	9 (19)	5 (20)	5 (17)	18 (27)	14 (18)
Non-squamous	28 (68)	39 (81)	20 (80)	24 (83)	48 (73)	63 (82)
Tumor PD-L1 expression^b^						
< 1%	0	0	25 (100)	29 (100)	25 (38)	29 (38)
≥ 1%	41 (100)	48 (100)	0	0	41 (62)	48 (62)
1–49%	14 (34)	25 (52)	0	0	14 (21)	25 (32)
≥ 50%	27 (66)	23 (48)	0	0	27 (41)	23 (30)

Data are *n* (%) unless otherwise indicated. ^a^Smoking status was defined by patient self-report. Current and former smokers reported the number of cigarettes smoked per day, and former smokers also reported the date they permanently stopped smoking. ^b^Determined by the PD-L1 IHC 28–8 pharmDx assay (Dako)

*ECOG PS* Eastern Cooperative Oncology Group performance status; *PD-L1* programmed death ligand 1

At database lock, all patients in both treatment arms had completed or discontinued treatment. In the tumor PD-L1 ≥ 1%, tumor PD-L1 < 1%, and the combined PD-L1 ≥ 1% and < 1% populations, the median (range) treatment durations were 6.0 (0–24.0), 4.2 (0–24.0), and 5.1 (0–24.0) months for nivolumab plus ipilimumab versus 4.2 (0–49.4), 2.3 (0–24.3), and 3.8 (0–49.4) months for chemotherapy, respectively (Table 2). Among 66 patients receiving nivolumab plus ipilimumab, 9 completed 2 years of study treatment (PD-L1 ≥ 1%, *n* = 8; PD-L1 < 1%, *n* = 1). Among patients in the combined PD-L1 ≥ 1% and < 1% population who had a PFS event, 67% (nivolumab plus ipilimumab) versus 85% (chemotherapy) received subsequent systemic anticancer therapies; 18% versus 81% received subsequent immunotherapy (Table 3).

**Table 2:** Duration of treatment and TFI^a^ in Japanese patients by tumor PD-L1 expression and in patients alive at 5 years (PD-L1 ≥ 1% and < 1%)

All treated patients	PD-L1 ≥ 1%		PD-L1 < 1%		PD-L1 ≥ 1% and < 1%	
	Nivolumab plus ipilimumab (*n* = 41)	Chemotherapy (*n* = 47)	Nivolumab plus ipilimumab (*n* = 25)	Chemotherapy (*n* = 29)	Nivolumab plus ipilimumab (*n* = 66)	Chemotherapy (*n* = 76)
Median duration of treatment^b^, months (range)	6.0 (0–24.0)	4.2 (0–49.4)	4.2 (0–24.0)	2.3 (0–24.3)	5.1 (0–24.0)	3.8 (0–49.4)
Median TFI^c^, months (95% CI)	9.7 (4.3–32.3)	1.8 (1.4–2.1)	2.6 (0.9–7.7)	1.5 (1.0–2.5)	5.7 (2.7–10.9)	1.7 (1.4–2.0)
TFI^c^, % (95% CI)						
1-year	46 (31–61)	11 (4–21)	24 (10–42)	3 (0–15)	38 (26–49)	8 (3–15)
2-year	39 (24–53)	5 (1–15)	12 (3–28)	0 (NA–NA)	29 (18–40)	3 (1–10)
3-year	32 (18–46)	5 (1–15)	12 (3–28)	0 (NA–NA)	24 (15–35)	3 (1–10)

Treated patients alive at 5 years	PD-L1 ≥ 1% and < 1%	
	Nivolumab plus ipilimumab (*n* = 27)	Chemotherapy (*n* = 20)
Median duration of treatment, months (range)	10.7 (0–24.0)	7.0 (2.0–49.4)
Median TFI^c^, months (95% CI)	NR (15.1–NR)	2.0 (1.2–5.3)
TFI^c^, % (95% CI)		
1-year	82 (61–92)	15 (4–34)
2-year	63 (42–78)	15 (4–34)
3-year	59 (39–75)	15 (4–34)

^a^TFI (defined as the time from last study dose to start of subsequent systemic therapy or death, whichever occurred first) analyses were conducted in patients who had discontinued study treatment. ^b^Median computed using Kaplan–Meier method. ^c^Based on Kaplan–Meier estimates

*CI* confidence interval; *NA* not applicable; *NR* not reached; *PD-L1* programmed death ligand 1; *TFI* treatment-free interval

**Table 3:** Subsequent therapy by tumor PD-L1 expression in Japanese patients with a PFS event per BICR and in patients alive at 5 years (PD-L1 ≥ 1% and < 1%)

Patients with a PFS event, *n* (%)	PD-L1 ≥ 1%		PD-L1 < 1%		PD-L1 ≥ 1% and < 1%	
	Nivolumab plus ipilimumab (*n* = 26)	Chemotherapy (*n* = 33)	Nivolumab plus ipilimumab (*n* = 19)	Chemotherapy (*n* = 19)	Nivolumab plus ipilimumab (*n* = 45)	Chemotherapy (*n* = 52)
Any	19 (73)	29 (88)	16 (84)	17 (89)	35 (78)	46 (88)
Radiotherapy	11 (42)	12 (36)	8 (42)	7 (37)	19 (42)	19 (37)
Surgery	1 (4)	1 (3)	1 (5)	0	2 (4)	1 (2)
Systemic therapy	15 (58)	28 (85)	15 (79)	16 (84)	30 (67)	44 (85)
Chemotherapy	14 (54)	20 (61)	15 (79)	10 (53)	29 (64)	30 (58)
Immunotherapy	4 (15)	27 (82)	4 (21)	15 (79)	8 (18)	42 (81)
PD-1 inhibitors	4 (15)	26 (79)	2 (11)	14 (74)	6 (13)	40 (77)
Nivolumab	2 (8)	22 (67)	1 (5)	12 (63)	3 (7)	34 (65)
PDR001	0	1 (3)	0	0	0	1 (2)
Pembrolizumab	2 (8)	5 (15)	1 (5)	2 (11)	3 (7)	7 (13)
PD-L1 inhibitors	1 (4)	3 (9)	3 (16)	1 (5)	4 (9)	4 (8)
Atezolizumab	0	3 (9)	2 (11)	1 (5)	2 (4)	4 (8)
Durvalumab	1 (4)	0	1 (5)	0	2 (4)	0
Targeted therapy	4 (15)	9 (27)	6 (32)	7 (37)	10 (22)	16 (31)
Experimental drugs	1 (4)	2 (6)	1 (5)	2 (11)	2 (4)	4 (8)

5-year survivors, *n* (%)	PD-L1 ≥ 1% and < 1%	
	Nivolumab plus ipilimumab (*n* = 27)	Chemotherapy (*n* = 20)
Any	13 (48)	18 (90)
Radiotherapy	7 (26)	4 (20)
Surgery	3 (11)	1 (5)
Systemic therapy	11 (41)	17 (85)
Chemotherapy	10 (37)	8 (40)
Immunotherapy	4 (15)	17 (85)
PD-1 inhibitors	2 (7)	15 (75)
Nivolumab	0	12 (60)
Pembrolizumab	2 (7)	3 (15)
PD-L1 inhibitors	3 (11)	3 (15)
Atezolizumab	2 (7)	3 (15)
Durvalumab	1 (4)	0
Targeted therapy	7 (26)	4 (20)
Experimental drugs	1 (4)	4 (20)

Percentages may not sum to 100% as patients may have received more than 1 type of subsequent therapy (defined as therapy started on or after first dosing date [or randomization date if the patient had not received study treatment])

*BICR* blinded independent review; *PD-1* programmed death-1*; PD-L1* programmed death ligand 1; *PFS* progression-free survival

### Efficacy outcomes

#### All randomized patients

**Fig. 1:**
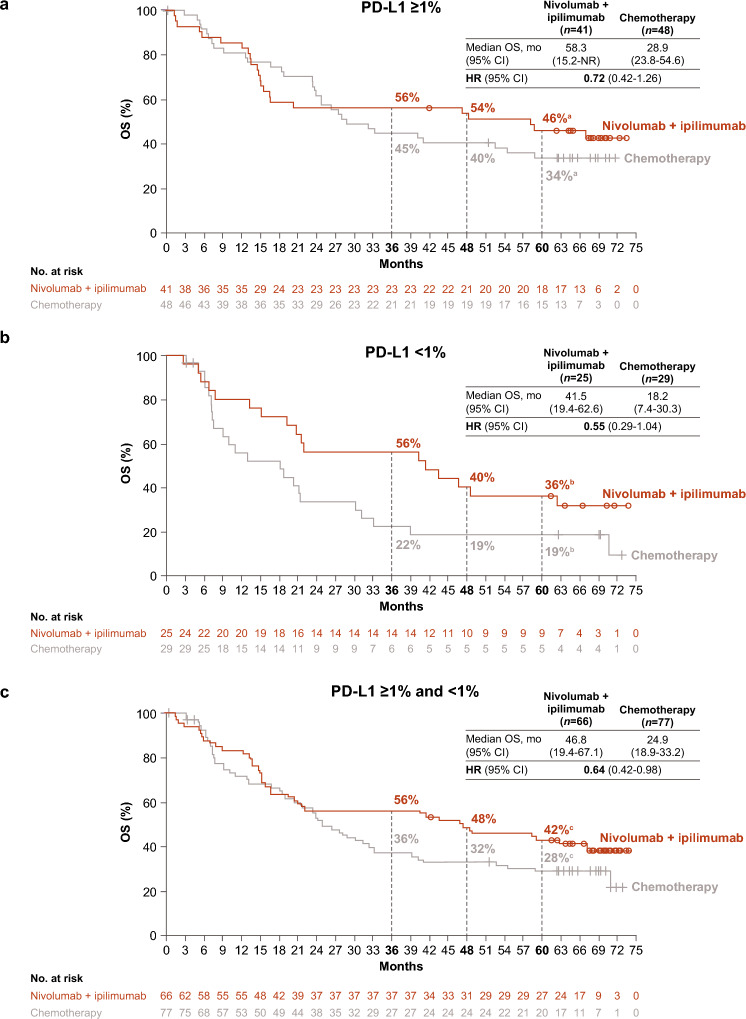
OS in Japanese patients with **a** tumor PD-L1 expression ≥ 1%, **b** tumor PD-L1 expression < 1%, and** c** tumor PD-L1 expression ≥ 1% and < 1%. ^a^95% CIs, 30%–60% (nivolumab plus ipilimumab) and 21%–47% (chemotherapy). ^b^95% CIs, 18%–54% (nivolumab plus ipilimumab) and 7%–35% (chemotherapy). ^c^95% CIs, 30%–54% (nivolumab plus ipilimumab) and 18%–39% (chemotherapy). *CI* confidence interval; *HR* hazard ratio; *OS* overall survival; *PD-L1* programmed death ligand 1

Among 89 Japanese patients with tumor PD-L1 ≥ 1%, median (95% CI) OS was 58.3 (15.2–not reached [NR]) months with nivolumab plus ipilimumab versus 28.9 (23.7–54.6) months with chemotherapy (HR 0.72; 95% CI, 0.42–1.26); 5-year OS (95% CI) rate was 46% (30%–60%) versus 34% (21%–47%) (Fig. 1A). Similar trends were observed with nivolumab plus ipilimumab versus chemotherapy in the tumor PD-L1 < 1% population (*n* = 54); median (95% CI) OS was 41.5 (19.4–62.6) versus 18.2 (7.4–30.3) months (HR 0.55; 95% CI, 0.29–1.04) and the 5-year OS (95% CI) rate was 36% (18%–54%) versus 19% (7%–35%) (Fig. 1B). Median (95% CI) OS in the combined PD-L1 ≥ 1% and < 1% population was 46.8 (19.4–67.1) versus 24.9 (18.9–33.2) months (HR 0.64, [95% CI 0.42–0.98]); 5-year OS (95% CI) rate was 42% (30%–54%) versus 28% (18%–39%) (Fig. 1C).

Durable OS benefit of nivolumab plus ipilimumab versus chemotherapy was also observed in patients with non-squamous (median [95% CI] OS: 58.3 [20.4–NR] vs 26.4 [20.4–33.4] months) or squamous (median [95% CI] OS: 29.1 [14.6–48.8] vs 9.2 [5.3–41.1] months) histology, regardless of tumor PD-L1 expression; however, sample sizes were small in the squamous subgroup (Supplementary Table 1).

**Fig. 2:**
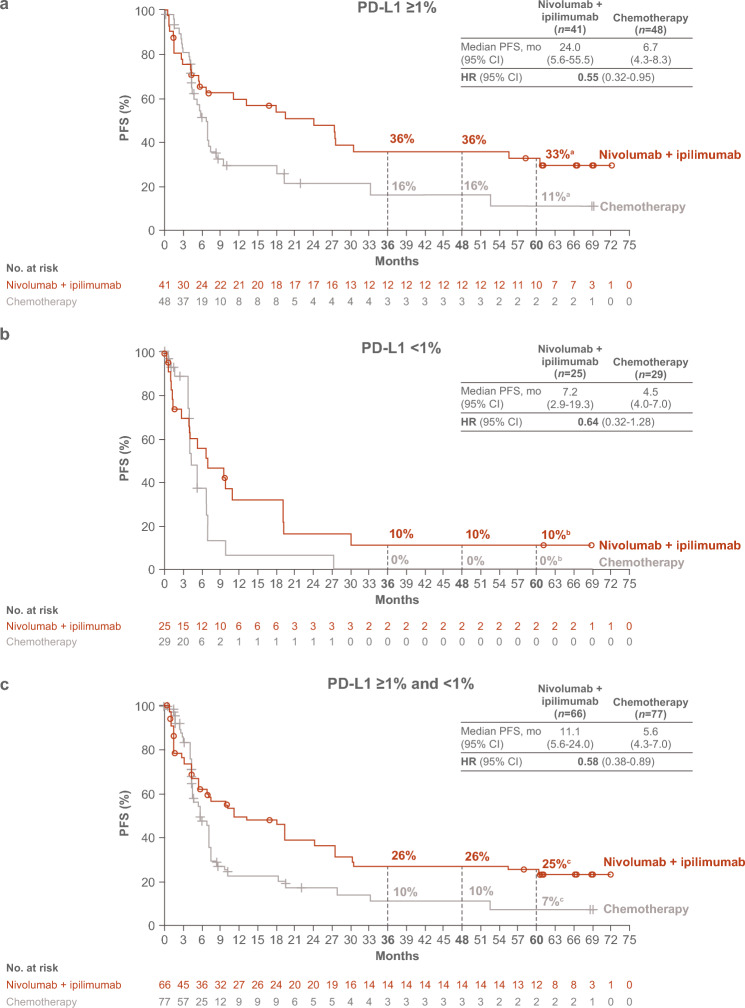
PFS in Japanese patients with **a** tumor PD-L1 expression ≥ 1%, **b** tumor PD-L1 expression < 1%, and** c** tumor PD-L1 expression ≥ 1% and < 1%. ^a^95% CIs, 18%–48% (nivolumab plus ipilimumab) and 2%–27% (chemotherapy). ^b^95% CIs, 2%–28% (nivolumab plus ipilimumab) and NA (chemotherapy). ^c^95% CIs, 14%–36% (nivolumab plus ipilimumab) and 1%–18% (chemotherapy). *CI* confidence interval; *HR* hazard ratio; *NA* not available*; PD-L1* programmed death ligand 1; *PFS* progression-free survival

Similar patterns of clinical benefit with nivolumab plus ipilimumab versus chemotherapy were observed for PFS, ORR, and DOR in both the tumor PD-L1 ≥ 1% and < 1% populations. In patients with tumor PD-L1 ≥ 1%, median (95% CI) PFS was 24.0 (5.6–55.5) months with nivolumab plus ipilimumab versus 6.7 (4.3–8.3) months with chemotherapy (HR 0.55; 95% CI, 0.32–0.95); 5-year PFS rate was 33% versus 11% (Fig. 2A). In patients with tumor PD-L1 < 1%, median PFS (95% CI) was 7.2 (2.9–19.3) months with nivolumab plus ipilimumab versus 4.5 (4.0–7.0) months with chemotherapy (HR 0.64; 95% CI, 0.32–1.28); 5-year PFS rate was 10% versus 0% (Fig. 2B). In the combined PD-L1 ≥ 1% and < 1% population, median (95% CI) PFS was 11.1 (5.6–24.0) months with nivolumab plus ipilimumab versus 5.6 (4.3–7.0) months with chemotherapy (HR 0.58; 95% CI, 0.38–0.89); 5-year PFS rate was 25% versus 7% (Fig. 2C). ORR was higher with nivolumab plus ipilimumab versus chemotherapy (63% vs 40%) in patients with tumor PD-L1 ≥ 1% but was comparable between treatment arms for patients with tumor PD-L1 < 1% (36% vs 31%) (Table 4). Median (95% CI) DOR was 59.1 (24.5–NR) months with nivolumab plus ipilimumab versus 7.1 (3.9–31.6) months with chemotherapy in the tumor PD-L1 ≥ 1% population, 17.3 (7.2–NR) versus 3.0 (2.6–5.6) months in the tumor PD-L1 < 1% population, and 29.0 (18.0–NR) versus 5.6 (3.1–7.1) months in the combined PD-L1 ≥ 1% and < 1% population (Table 4). Of the confirmed responders, 40% (nivolumab plus ipilimumab) versus 6% (chemotherapy) had ongoing responses for ≥ 5 years.

**Table 4:** Tumor response and DOR in Japanese patients by tumor PD-L1 expression and in patients alive at 5 years (PD-L1 ≥ 1% and < 1%)

All randomized patients	PD-L1 ≥ 1%		PD-L1 < 1%		PD-L1 ≥ 1% and < 1%	
	Nivolumab plus ipilimumab (*n* = 41)	Chemotherapy (*n* = 48)	Nivolumab plus ipilimumab (*n* = 25)	Chemotherapy (*n* = 29)	Nivolumab plus ipilimumab (*n* = 66)	Chemotherapy (*n* = 77)
ORR, %	63	40	36	31	53	36
Best overall response, *n* (%)						
Complete response	10 (24)	2 (4)	2 (8)	0	12 (18)	2 (3)
Partial response	16 (39)	17 (35)	7 (28)	9 (31)	23 (35)	26 (34)
Stable disease	7 (17)	24 (50)	8 (32)	17 (59)	15 (23)	41 (53)
Progressive disease	7 (17)	4 (8)	6 (24)	2 (7)	13 (20)	6 (8)
Not determined	1 (2)	1 (2)	2 (8)	1 (3)	3 (5)	2 (3)
Median DOR^a^, months (95% CI)	59.1 (24.5–NR)	7.1 (3.9–31.6)	17.3 (7.2–NR)	3.0 (2.6–5.6)	29.0 (18.0–NR)	5.6 (3.1–7.1)
Ongoing response at ≥ 5 years^b^, %	46	9	25	NA	40	6

Patients alive at 5 years	PD-L1 ≥ 1% and < 1%	
	Nivolumab plus ipilimumab (*n* = 27)	Chemotherapy (*n* = 20)
ORR, %	85	45
Best overall response, *n* (%)		
Complete response	12 (44)	1 (5)
Partial response	11 (41)	8 (40)
Stable disease	4 (15)	9 (45)
Progressive disease	0	2 (10)
Not determined	0	0
Median DOR^a^, months (95% CI)	NR (24.9–NR)	6.9 (2.7–31.6)
Ongoing response^b^ at ≥ 5 years, %	56	17

^a^Computed using Kaplan–Meier method. ^b^Based on Kaplan–Meier estimates of DOR

*CI* confidence interval; *DOR* duration of response; *NA* not applicable; *NR* not reached; *ORR* objective response rate; *PD-L1* programmed death ligand 1

PFS2 benefit with nivolumab plus ipilimumab versus chemotherapy was observed in the tumor PD-L1 ≥ 1% (HR 0.56; 95% CI, 0.33–0.94) and tumor PD-L1 < 1% (HR 0.52; 95% CI, 0.29–0.93) populations (Supplementary Fig. 2). TFI, a potential indicator of patient experience [26–29], was assessed by tumor PD-L1 expression (Table 2). Among patients who discontinued study treatment (for any reason), 32% (PD-L1 ≥ 1%) and 12% (PD-L1 < 1%) of patients in the nivolumab plus ipilimumab arm versus 5% (PD-L1 ≥ 1%) and 0% (PD-L1 < 1%) in the chemotherapy arm were estimated to remain alive and treatment-free for ≥ 3 years after treatment discontinuation.

#### Patients alive at 5 years

**Fig. 3:**
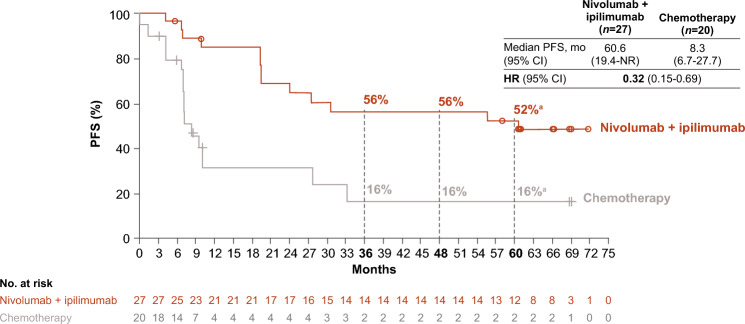
PFS in Japanese patients alive at 5 years (PD-L1 ≥ 1% and < 1%). ^a^95% CIs, 32%–70% (nivolumab plus ipilimumab) and 3%–38% (chemotherapy). *CI* confidence interval; *HR* hazard ratio; *NR* not reached; *PD-L1* programmed death ligand 1; *PFS* progression-free survival

In the combined PD-L1 ≥ 1% and < 1% population, 47 Japanese patients were alive at 5 years (nivolumab plus ipilimumab: *n* = 27; chemotherapy: *n* = 20); baseline characteristics of patients are shown in Supplementary Table 2. Median (range) treatment duration was 10.7 (0–24.0) months for nivolumab plus ipilimumab and 7.0 (2.0–49.4) months for chemotherapy (Table 2), with a median (range) of 24 (1–52) nivolumab doses and 6 (1–18) ipilimumab doses received in the nivolumab plus ipilimumab arm. PFS benefit was observed with nivolumab plus ipilimumab versus chemotherapy; median (95% CI) PFS was 60.6 (19.4–NR) versus 8.3 (6.7–27.7) months (Fig. 3). ORR was 85% (nivolumab plus ipilimumab) versus 45% (chemotherapy), with 56% versus 17% of responders having ongoing responses for ≥ 5 years; median (95% CI) DOR was NR versus 6.9 (2.7–31.6) months (Table 4).

At the 5-year cut-off, 59% (95% CI, 39%–75%) of patients in the nivolumab plus ipilimumab arm versus 15% (95% CI, 4%–34%) in the chemotherapy arm were off treatment without receiving any subsequent therapy (Table 2); 15% versus 85% of patients had received subsequent immunotherapy (Table 3). Median (95% CI) TFI was NR with nivolumab plus ipilimumab versus 2.0 (1.2–5.3) months with chemotherapy; the 3-year TFI rate was 59% versus 15% (Table 2).

#### Patients who discontinued study treatment due to TRAEs

**Fig. 4:**
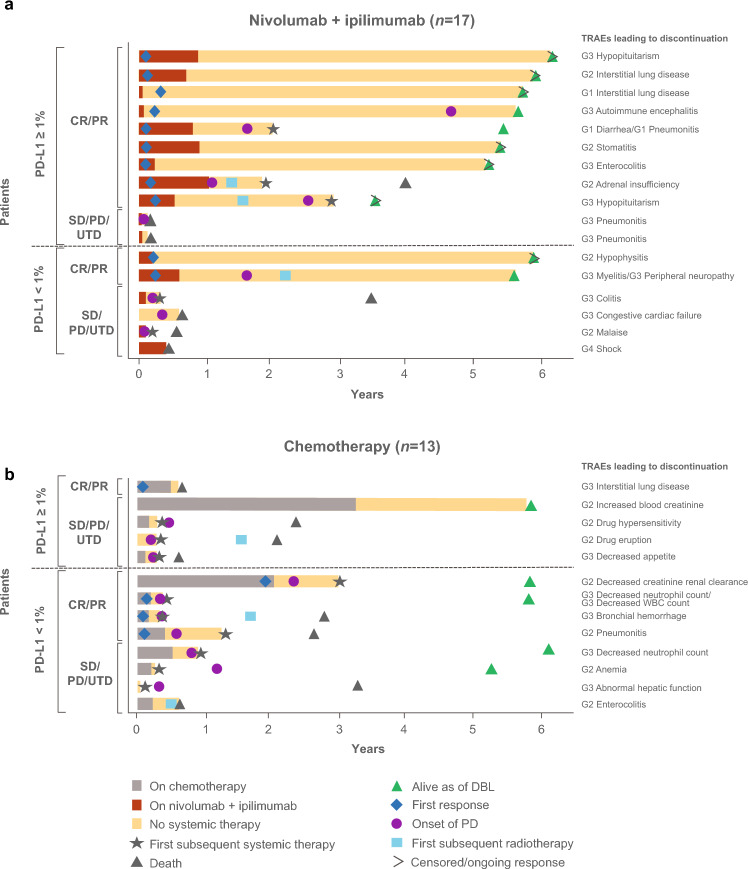
Swimmer plot of patients who discontinued treatment due to TRAEs^a^ among Japanese patients treated with **a** nivolumab plus ipilimumab or **b** chemotherapy. ^a^Includes events reported between first dose and 30 days after last dose of study therapy in patients who discontinued treatment due to study drug toxicity. *CR* complete response; *DBL* database lock; *G1* Grade 1; *G2* Grade 2; *G3* Grade 3; *G4* Grade 4; *IPI* ipilimumab; *NIVO* nivolumab; *PD* progressive disease*; PD-L1* programmed death ligand 1; *PR* partial response; *SD* stable disease; *TRAE* treatment-related adverse event; *UTD* undetermined; *WBC* white blood cell

In the combined PD-L1 ≥ 1% and < 1% population, TRAEs led to the discontinuation of all study drugs in 17 (26%) Japanese patients treated with nivolumab plus ipilimumab and 13 (17%) treated with chemotherapy (Fig. 4). Median (range) treatment duration was 2.8 (0–12.6) months with nivolumab plus ipilimumab and 2.2 (0–38.9) months with chemotherapy (Supplementary Table 3). Median OS was NR with nivolumab plus ipilimumab versus 33.2 (95% CI, 7.7–NR) months with chemotherapy; 5-year OS rate was 58% versus 38% (Table 5). Median PFS after treatment discontinuation was 54.3 months versus 2.0 months. ORR was 65% (nivolumab plus ipilimumab) versus 38% (chemotherapy) and median DOR after treatment discontinuation was NR versus 1.5 (95% CI, 0.1–NR) months; 66% of responding patients in the nivolumab plus ipilimumab arm had responses lasting ≥ 5 years, whereas no patients in the chemotherapy arm were estimated to remain in response. The 3-year TFI rate was 47% with nivolumab plus ipilimumab; no patients were estimated to be treatment-free in the chemotherapy arm (Supplementary Table 3).

**Table 5:** Efficacy outcomes in Japanese patients who discontinued study treatment due to TRAEs^a^ (PD-L1 ≥ 1% and < 1%)

	Nivolumab plus ipilimumab (*n* = 17)	Chemotherapy (*n* = 13)
Median OS^b^, months (95% CI)	NR	33.2 (7.7–NR)
5-year OS rate^b^, %	58	38
Median PFS after discontinuation^b^, months (95% CI)	54.3 (0.8–NR)	2.0 (0.8–NR)
ORR, %	65	38
Median DOR^c^ after discontinuation, months (95% CI)	NR	1.5 (0.1–NR)
Ongoing response^d^ at ≥ 3 years after discontinuation, % (95% CI)	79 (38–94)	NA

^a^Includes patients with TRAEs reported between first dose and 30 days after last dose of study therapy who discontinued treatment due to study drug toxicity. ^b^Based on Kaplan–Meier estimates. ^c^Computed using Kaplan–Meier method. ^d^Based on Kaplan–Meier estimates of DOR

*CI* confidence interval; *DOR* duration of response; *NA* not applicable; *NR* not reached; *ORR* objective response rate; *OS* overall survival; *PD-L1* programmed death ligand 1; *PFS* progression-free survival; *TRAE* treatment-related adverse event

### Safety

No new TRAEs were reported with nivolumab plus ipilimumab since the prior report [21], as all Japanese patients have been off treatment for ≥ 3 years (Supplementary Table 4). In the combined PD-L1 ≥ 1% and < 1% population, the most common (≥ 10%) any-grade IMAEs with nivolumab plus ipilimumab were rash (47%), diarrhea/colitis (18%), adrenal insufficiency, hypothyroidism/thyroiditis, and hypophysitis (12% each), and hyperthyroidism and pneumonitis (11% each); the most common (≥ 5%) grade 3–4 IMAEs were hypophysitis (8%), adrenal insufficiency, diabetes mellitus, and rash (6% each), and diarrhea/colitis, pneumonitis, and hepatitis (5% each) (Table 6).

**Table 6:** Incidence and times to onset and resolution of IMAEs in Japanese patients treated with nivolumab plus ipilimumab

	Nivolumab plus ipilimumab (PD-L1 ≥ 1% and < 1%) (*n* = 66)				
IMAE^a^, *n* (%)	Any grade	Grade 3–4	Median time to onset, months (range)	Median^b^ time to resolution, months (range)	Resolved IMAEs^c^, *n*/*N* (%)
Non-endocrine IMAEs					
Rash	31 (47)	4 (6)	0.3 (0.1–12.2)	3.4 (0.2 to 67.2 +)	22/31 (71)
Diarrhea/colitis	12 (18)	3 (5)	4.8 (0.2–14.6)	4.0 (0.3 to 61.1 +)	11/12 (92)
Pneumonitis	7 (11)	3 (5)	0.7 (0.1–19.5)	7.9 (0.9 + to 13.4)	4/7 (57)
Hepatitis	5 (8)	3 (5)	2.5 (0.3–8.9)	1.5 (0.5 to 5.5 +)	4/5 (80)
Hypersensitivity	1 (2)	0	< 1	< 1	1/1 (100)
Nephritis and renal function	0	0	NA	NA	NA
Endocrine IMAEs					
Adrenal insufficiency	8 (12)	4 (6)	4.1 (1.0–14.8)	NR (4.2 to 67.3 +)	1/8 (12)
Hypothyroidism/thyroiditis	8 (12)	1 (2)	2.3 (1.0–4.2)	NR (9.0 + to 66.8 +)	1/8 (12)
Hypophysitis	8 (12)	5 (8)	2.6 (2.0–10.7)	NR (0.5 to 67.8 +)	1/8 (12)
Hyperthyroidism	7 (11)	0	2.3 (1.4–4.2)	1.9 (0.7 to 3.9)	7/7 (100)
Diabetes mellitus	4 (6)	4 (6)	4.9 (4.6–13.6)	NR (8.2 + to 66.1 +)	0/4 (0)

+ indicates a censored value. ^a^Includes adverse events considered as potential immune-mediated events by investigator occurring within 100 days of last dose of study drug regardless of causality and treated with immune-modulating medication, with the exception of endocrine events (adrenal insufficiency, hypophysitis, hypothyroidism/thyroiditis, hyperthyroidism, and diabetes mellitus), which were included in the analysis regardless of treatment since these events are often managed without immunosuppression. ^b^From Kaplan–Meier estimation. ^c^Events without a stop date or where stop date was death date, as well as grade 5 events, were considered unresolved. Events without worsening from baseline were excluded

*CI* confidence interval; *IMAE* immune-mediated adverse event; *NA* not applicable, *NR* not reached; *PD-L1* programmed death ligand 1

**Fig. 5:**
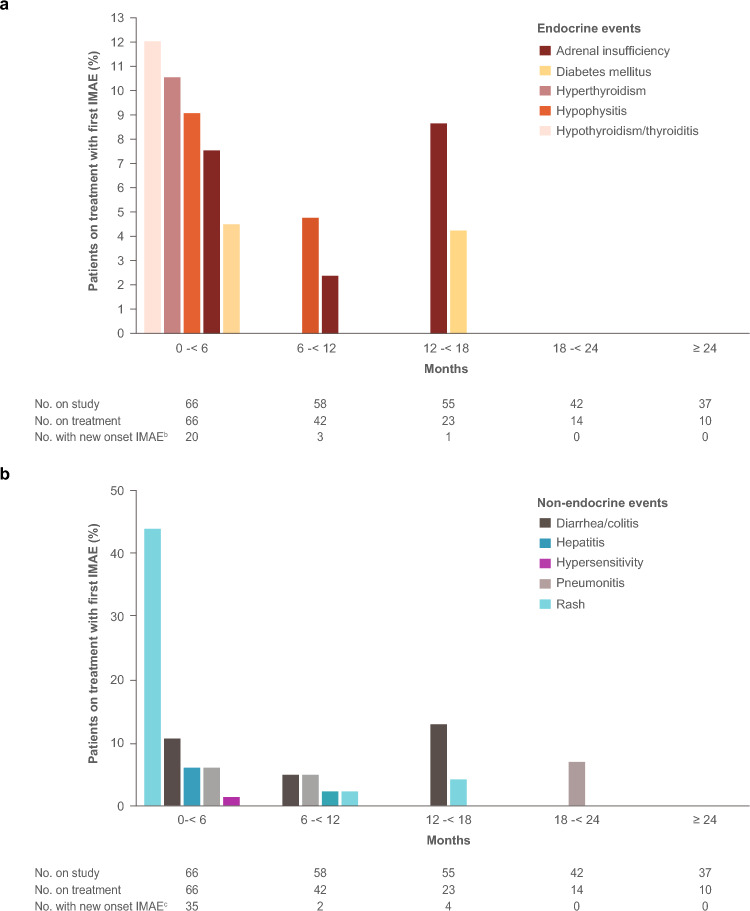
First incidence of **a** endocrine and **b** non-endocrine IMAEs^a^ over time in Japanese patients treated with nivolumab plus ipilimumab (*n* = 66). In patients who experienced multiple IMAEs, the first incidence of each IMAE was reported separately. ^a^Includes adverse events considered as potential immune-mediated events by investigator occurring within 100 days of last dose of study drug regardless of causality and treated with immune-modulating medication, except for endocrine events (adrenal insufficiency, hypophysitis, hypothyroidism/thyroiditis, hyperthyroidism, and diabetes mellitus), which were included in the analysis regardless of treatment since these events are often managed without immunosuppression. ^b^Represents the number of patients newly experiencing an onset of any endocrine IMAE. ^c^Represents the number of patients newly experiencing an onset of any non-endocrine IMAE. *IMAE* immune-mediated adverse event

Most IMAEs occurred within the first 6 months of nivolumab plus ipilimumab treatment, with 20 (30%) and 35 (53%) patients newly experiencing an onset of an endocrine or non-endocrine IMAE during this time, respectively (Table 6; Fig. 5; Supplementary Fig. 3). The frequency of new onset of IMAEs was considerably reduced over time, with 3 and 2 patients experiencing new onset of endocrine and non-endocrine IMAEs, respectively, at 6–12 months and 1 and 4 patients at 12–18 months. No patient had new onset of IMAEs beyond 18 months of treatment (Fig. 5). Most non-endocrine IMAEs were resolved, with the median time to resolution ranging from < 1 month (hypersensitivity) to 7.9 months (pneumonitis) (Table 6). Systemic corticosteroids were primarily used for the management of most IMAEs in patients treated with nivolumab plus ipilimumab, with median treatment duration ranging from 0.1 (hypersensitivity) to 3.9 weeks (adrenal insufficiency) (Supplementary Table 5).

## Discussion

The results of this subanalysis in Japanese patients from the CheckMate 227 Part 1 study represent the longest survival follow-up reported to date for phase 3 studies evaluating first-line combination immunotherapy for metastatic NSCLC with tumor PD-L1 ≥ 1% or < 1%. With a 5-year minimum follow-up, clinically meaningful OS benefit was maintained with nivolumab plus ipilimumab versus chemotherapy in Japanese patients regardless of tumor PD-L1 expression, despite a high rate of subsequent immunotherapy received in the chemotherapy arm (81%) in the combined PD-L1 ≥ 1% and < 1% population of patients with a PFS event. The results were consistent with the 3-year follow-up analysis in Japanese patients [21].

PFS and DOR benefit was maintained with nivolumab plus ipilimumab at 5 years. Additionally, patients in the combined PD-L1 ≥ 1% and < 1% population who were alive at 5 years had a higher 3-year TFI rate with nivolumab plus ipilimumab versus chemotherapy, with 59% versus 15% of patients receiving no subsequent systemic therapy at the 5-year time point, suggesting the long-term durable benefit of this first-line immunotherapy combination in Japanese patients. Among patients who discontinued nivolumab plus ipilimumab treatment within 2 years due to TRAEs, 58% remained alive for ≥ 5 years, with 47% of patients receiving no subsequent therapy within 3 years of discontinuation.

Recent studies in melanoma and other cancers have demonstrated that TFI, during which patients typically experience clinically stable disease while remaining treatment-free, is associated with improved quality of life [26–29]. In the randomized global population of CheckMate 227, two-thirds of the 5-year survivors treated with nivolumab plus ipilimumab remained treatment-free through the 5-year landmark, and their quality of life was similar to that of the US general population [11, 30]. In this subanalysis, the high 3-year TFI rate in 5-year survivors who received nivolumab plus ipilimumab is consistent with that of the randomized global population [11], and also suggests long-term durable benefit of this dual immunotherapy regimen in Japanese patients.

No new safety signals were reported in this subanalysis after long-term follow-up (≥ 5 years). TRAEs reported in Japanese patients at 5 years were consistent with the 3-year data [21]. Most IMAEs occurred within the first 6 months of nivolumab plus ipilimumab treatment; only 1 and 4 Japanese patients experienced new onset of endocrine and non-endocrine IMAEs, respectively, after 12 months. Numerically higher incidences of some IMAEs were observed in Japanese patients versus the randomized global population [11], including rash (47% vs 20%), diarrhea/colitis (18% vs 8%), adrenal insufficiency (12% vs 4%), hypophysitis (12% vs 4%), and diabetes mellitus (6% vs 1%); however, most events were managed with systemic corticosteroids.

This exploratory subanalysis was not statistically powered to conduct statistical testing for comparisons between treatment arms. The subanalysis was also limited by small sample sizes for the patient subgroups. The findings in Japanese patients, however, were consistent with results in the randomized global population and aligned with findings from other immunotherapy-based treatments in this subpopulation [11, 21, 31–33]. Ongoing observational studies in Japanese patients with metastatic NSCLC could provide insight into the use of first-line nivolumab plus ipilimumab in clinical practice in Japan [34].

In conclusion, at ≥ 5-year follow-up, first-line nivolumab plus ipilimumab provided long-term efficacy benefits versus chemotherapy in Japanese patients with metastatic NSCLC, regardless of tumor PD-L1 expression. Consistent with the long-term findings in the randomized global population, these data continue to support the use of nivolumab plus ipilimumab as first-line treatment for patients with metastatic NSCLC in Japan.

### Supplementary Information

Below is the link to the electronic supplementary material.

Supplementary file1 (DOCX 543 KB)

## Data Availability

Data are available upon reasonable request. BMS policy on data sharing may be found at https://www.bms.com/researchers-and-partners/independent-research/data-sharing-request-process.html.
