# Correction to: First-line nivolumab plus ipilimumab in metastatic non-small cell lung cancer: 5-year outcomes in Japanese patients from CheckMate 227 Part 1

**DOI:** 10.1007/s10147-023-02408-9

**Published:** 2023-09-09

**Authors:** Makoto Nishio, Yuichiro Ohe, Satoshi Ikeda, Toshihide Yokoyama, Hidetoshi Hayashi, Tatsuro Fukuhara, Yuki Sato, Hiroshi Tanaka, Katsuyuki Hotta, Shunichi Sugawara, Haruko Daga, Isamu Okamoto, Kazuo Kasahara, Tateaki Naito, Li Li, Ravi G. Gupta, Judith Bushong, Hideaki Mizutani

**Affiliations:** 1 Cancer Institute Hospital, Japanese Foundation for Cancer Research, 3-8-31, Ariake, Koto, Tokyo, 135-8550 Japan; 2 National Cancer Center Hospital, 5-1-1 Tsukiji, Chuo-ku, Tokyo, 104-0045 Japan; 3 Kanagawa Cardiovascular and Respiratory Center, 6-16-1 Tomiokahigashi, Kanazawa-ku, Yokohama-shi, Kanagawa, 236-0051 Japan; 4 Kurashiki Central Hospital, 1-1-1 Miwa, Kurashiki, Okayama 710-8602 Japan; 5 Kindai University Hospital, 3-4-1 Kowakae, Higashiosaka, Osaka 577-8502 Japan; 6 Miyagi Cancer Center, 47-1 Nodayama, Shiote, Medeshima, Natori, Miyagi 981-1293 Japan; 7 Department of Respiratory Medicine, Kobe City Medical Center General Hospital, 2-1-1 Minatojima-Minamimachi, Chuo-ku, Kobe, 650-0047 Japan; 8 Niigata Cancer Center Hospital, 2-15-3 Kawagishi-cho, Chuo-ku, Niigata, 951-8566 Japan; 9 Okayama University Hospital, 2-5-1 Shikata-cho, Kita-ku, Okayama, 700-8558 Japan; 10 Sendai Kousei Hospital, 4-15 Hirose-machi, Aoba-ku, Sendai, Miyagi 980-0873 Japan; 11 Osaka City General Hospital, 2-13-22 Miyakojima Hondori, Miyakojima Ward, Osaka, 534-0021 Japan; 12 Kyushu University Hospital, 3-1-1 Maidashi, Higashi-ku, Fukuoka, 812-8582 Japan; 13 Kanazawa University Hospital, 13-1 Takaramachi, Kanazawa, Ishikawa 920-8641 Japan; 14 Shizuoka Cancer Center, 1007 Shimonagakubo, Nagaizumi-Cho, Sunto-Gun, Shizuoka 411-8777 Japan; 15 Bristol Myers Squibb, Princeton, NJ USA; 16 Saitama Cancer Center, 780 Oaza Komuro, Ina Machi, Kita-Adachi-gun, Saitama, 362-0806 Japan

**Correction to: International Journal of Clinical Oncology** 10.1007/s10147-023-02390-2

The article, First‑line nivolumab plus ipilimumab in metastatic non‑small cell lung cancer: 5‑year outcomes in Japanese patients from CheckMate 227 Part 1 written by Makoto Nishio, Yuichiro Ohe, Satoshi Ikeda, Toshihide Yokoyama, Hidetoshi Hayashi, Tatsuro Fukuhara, Yuki Sato, Hiroshi Tanaka, Katsuyuki Hotta, Shunichi Sugawara, Haruko Daga, Isamu Okamoto, Kazuo Kasahara, Tateaki Naito, Li Li, Ravi G. Gupta, Judith Bushong, Hideaki Mizutani was originally published Online First without Open Access.

With the author(s)’ decision to opt for Open Choice the copyright of the article changed on September 2, 2023 to © Author(s) 2023 and the article is forthwith distributed under a Creative Commons Attribution 4.0 International License, which permits use, sharing, adaptation, distribution and reproduction in any medium or format, as long as you give appropriate credit to the original author(s) and the source, provide a link to the Creative Commons licence, and indicate if changes were made. The images or other third party material in this article are included in the article’s Creative Commons licence, unless indicated otherwise in a credit line to the material. If material is not included in the article’s Creative Commons licence and your intended use is not permitted by statutory regulation or exceeds the permitted use, you will need to obtain permission directly from the copyright holder. To view a copy of this licence, visit http://creativecommons.org/licenses/by/4.0.

The original article has been corrected.

