# Supplementary material for: First-line nivolumab plus ipilimumab in metastatic non-small cell lung cancer: 5-year outcomes in Japanese patients from CheckMate 227 Part 1
Source: Int J Clin Oncol. 2023 Aug 7;28(10):1354–68. doi: 10.1007/s10147-023-02390-2 (PMC10542710; doi:10.1007/s10147-023-02390-2)

**Supplementary materials (online only)**

**First-line nivolumab plus ipilimumab in metastatic non-small cell lung cancer: 5-year outcomes in Japanese patients from CheckMate 227 Part 1**

Makoto Nishio, Yuichiro Ohe, Satoshi Ikeda, Toshihide Yokoyama, Hidetoshi Hayashi, Tatsuro Fukuhara, Yuki Sato, Hiroshi Tanaka, Katsuyuki Hotta, Shunichi Sugawara, Haruko Daga, Isamu Okamoto, Kazuo Kasahara, Tateaki Naito, Li Li, Ravi G. Gupta, Judith Bushong, Hideaki Mizutani

**Journal:** International Journal of Clinical Oncology

**Corresponding author**:

Makoto Nishio, MD, PhD

Cancer Institute Hospital, Japanese Foundation for Cancer Research,

3-8-31, Ariake, Koto, Tokyo, 135-8550, Japan

Email: [mnishio@jfcr.or.jp](mailto:mnishio@jfcr.or.jp)

**Supplementary methods**

Treatment-related adverse events (TRAEs) were assessed by the investigator and collected within 30 days after last dose of study drug. Immune-mediated adverse events (IMAEs) were defined as potential immune-mediated events occurring within 100 days of last dose of study drug regardless of causality and treated with immune-modulating medication, except for endocrine events, which were included in the analysis regardless of treatment since these events are often managed without immunosuppression.

**Supplementary Table 1** OS by histology in Japanese patients and tumor PD-L1 subgroups

|  | **PD-L1 ≥ 1%** | | **PD-L1 < 1%** | | **PD-L1 ≥ 1% and < 1%** | |
| --- | --- | --- | --- | --- | --- | --- |
|  | **Nivolumab plus ipilimumab** | **Chemotherapy** | **Nivolumab plus ipilimumab** | **Chemotherapy** | **Nivolumab plus ipilimumab** | **Chemotherapy** |
| **Non-squamous, *n*** | **28** | **39** | **20** | **24** | **48** | **63** |
| Median OS^a^, months  (95% CI) | NR  (15.2–NR) | 32.8  (24.8–NR) | 31.2  (13.3–NR) | 18.2  (7.6–21.5) | 58.3  (20.4–NR) | 26.4  (20.4–33.4) |
| HR^b^ (95% CI) | 0.70 (0.35–1.37) | | 0.53 (0.26–1.08) | | 0.66 (0.41–1.08) | |
| 5-year OS rate^a^, % | 57 | 36 | 40 | 17 | 50 | 29 |
| **Squamous, *n*** | **13** | **9** | **5** | **5** | **18** | **14** |
| Median OS^a^, months  (95% CI) | 16.6  (12.2–58.9) | 9.2  (2.9–NR) | 43.6  (7.9–NR) | 20.0  (5.3–NR) | 29.1  (14.6–48.8) | 9.2  (5.3–41.1) |
| HR^b^ (95% CI) | ND | | ND | | 0.74 (0.33–1.68) | |
| 5-year OS rate^a^, % | 23 | 22 | 20 | 25 | 22 | 23 |

^a^Based on Kaplan-Meier estimates. ^b^HRs are based on an unstratified Cox proportional hazards model; HR is not displayed when there are ≤ 10 patients in each treatment arm*.*

*CI* confidence interval; *HR* hazard ratio; *ND* not displayed; *NR* not reached; *OS* overall survival; *PD-L1* programmed death ligand 1

**Supplementary Table 2** Baseline characteristics of Japanese patients alive at 5 years

|  | **PD-L1 ≥ 1% and < 1%** | |
| --- | --- | --- |
|  | **Nivolumab**  **plus**  **ipilimumab**  **(*n*=27)** | **Chemotherapy**  **(*n*=20)** |
| Age, median (range), years, | 65 (42–78) | 66 (41–78) |
| Age category, years |  |  |
| < 65 | 13 (48) | 9 (45) |
| ≥ 65 to < 75 | 12 (44) | 10 (50) |
| ≥ 75 | 2 (7) | 1 (5) |
| Female | 4 (15) | 7 (35) |
| ECOG PS |  |  |
| 0 | 11 (41) | 12 (60) |
| 1 | 16 (59) | 8 (40) |
| Smoking status^a^ |  |  |
| Current/former smoker | 26 (96) | 16 (80) |
| Never-smoker | 1 (4) | 4 (20) |
| Histology |  |  |
| Squamous | 4 (15) | 3 (15) |
| Non-squamous | 23 (85) | 17 (85) |
| Tumor PD-L1 expression |  |  |
| < 1% | 9 (33) | 5 (25) |
| ≥ 1% | 18 (67) | 15 (75) |
| 1–49% | 3 (11) | 7 (35) |
| ≥ 50% | 15 (56) | 8 (40) |

Data are n (%) unless otherwise indicated. ^a^Smoking status was defined by patient self-report. Current and former smokers reported the number of cigarettes smoked per day, and former smokers also reported the date they permanently stopped smoking.

*ECOG PS* Eastern Cooperation Oncology Group performance status; *PD-L1* programmed death ligand 1.

**Supplementary Table 3** Duration of treatment and TFI^a^ in Japanese patients who discontinued study treatment due to TRAEs^b^

|  | **PD-L1 ≥ 1% and < 1%** | |
| --- | --- | --- |
|  | **Nivolumab plus ipilimumab**  **(*n*=17)** | **Chemotherapy**  **(*n*=13)** |
| Median duration of treatment, months (range) | 2.8  (0–12.6) | 2.2  (0–38.9) |
| Median TFI^c^, months  (95% CI) | 28.2  (1.0–NR) | 2.5  (1.3–4.9) |
| TFI rates, % (95% CI) |  |  |
| 1-year | 59 (32–78) | 8 (<1–29) |
| 2-year | 53 (28–73) | 8 (<1–29) |
| 3-year | 47 (23–68) | NA |

^a^TFI (defined as the time from last study dose to start of subsequent systemic therapy or death, whichever occurred first) analyses were conducted in patients who had discontinued study treatment. ^b^Includes patients with TRAEs reported between first dose and 30 days after last dose of study therapy who discontinued treatment due to study drug toxicity. ^c^Median computed using Kaplan-Meier method.

*CI* confidence interval; *NA* not applicable; *PD-L1* programmed death ligand 1; *TFI* treatment-free interval; *TRAE* treatment-related adverse event.

**Supplementary Table 4** Summary of TRAEs in all treated Japanese patients (PD-L1 ≥ 1% and < 1%)

| ***n* (%)** | **Nivolumab plus ipilimumab**  **(*n*=66)** | | **Chemotherapy  (*n*=76)** | |
| --- | --- | --- | --- | --- |
|  | **Any grade** | **Grade 3–4** | **Any grade** | **Grade 3–4** |
| Any TRAE^a^, | 63 (95) | 36 (55) | 73 (96) | 36 (47) |
| Reported in ≥ 15% patients |  |  |  |  |
| Pyrexia | 21 (32) | 1 (2) | 2 (3) | 0 |
| Maculopapular rash | 17 (26) | 3 (5) | 7 (9) | 0 |
| Diarrhea | 14 (21) | 0 | 8 (11) | 0 |
| Decreased appetite | 12 (18) | 1 (2) | 35 (46) | 3 (4) |
| Pruritus | 10 (15) | 0 | 1 (1) | 0 |
| AST increased | 10 (15) | 1 (2) | 11 (14) | 0 |
| Malaise | 7 (11) | 0 | 18 (24) | 0 |
| ALT increased | 7 (11) | 1 (2) | 15 (20) | 0 |
| Anemia | 6 (9) | 2 (3) | 25 (33) | 9 (12) |
| Constipation | 5 (8) | 0 | 45 (59) | 1 (1) |
| Nausea | 4 (6) | 1 (2) | 39 (51) | 1 (1) |
| Neutrophil count decreased | 3 (5) | 0 | 33 (43) | 15 (20) |
| Dysgeusia | 3 (5) | 0 | 12 (16) | 0 |
| Platelet count decreased | 1 (2) | 0 | 19 (25) | 4 (5) |
| Hiccups | 0 | 0 | 25 (33) | 0 |
| WBC count decreased | 0 | 0 | 17 (22) | 6 (8) |
| Treatment-related serious AEs | 37 (56) | 28 (42) | 13 (17) | 7 (9) |
| TRAEs leading to treatment discontinuation^b^ | 19 (29) | 11 (17) | 14 (18) | 6 (8) |
| Treatment-related deaths | 1 (2)^c^ | | 1 (1)^c^ | |

^a^Included events reported between first dose and 30 days after last dose of study therapy. ^b^These events included TRAEs leading to discontinuation of ipilimumab or both study drugs; discontinuation of nivolumab without discontinuation of ipilimumab was not permitted. ^c^Nivolumab plus ipilimumab: shock (*n*=1); chemotherapy: interstitial lung disease (*n*=1).

*AE* adverse event; *ALT* alanine transaminase; *AST* aspartate transaminase; *PD-L1* programmed death ligand 1; *TRAE* treatment-related adverse event; *WBC* white blood cell

**Supplementary Table 5** Systemic corticosteroid (≥ 40 mg/day) medication use for the management of IMAEs^a^ in Japanese patients treated with nivolumab plus ipilimumab (PD-L1 ≥ 1% and < 1%)

|  | **Nivolumab plus ipilimumab**  **(PD-L1 ≥ 1% and < 1%)**  **(*n*=66)** | | |
| --- | --- | --- | --- |
|  | **Corticosteroid ≥ 40 mg/day^b^** | **Median (range) duration of corticosteroid**  **≥ 40 mg/day, weeks** | **Corticosteroid + IMM^b^** |
| **Non-endocrine IMAE** |  |  |  |
| Rash | 3/31 (10) | 0.3 (0.3–0.4) | 0/31 (0) |
| Diarrhea/colitis | 7/12 (58) | 2.9 (0.4–10.1) | 3/12 (25) |
| Pneumonitis | 7/7 (100) | 2.6 (0.9–10.0) | 0/7 (0) |
| Hepatitis | 5/5 (100) | 3.1 (0.7–8.4) | 1/5 (20) |
| Hypersensitivity | 1/1 (100) | 0.1 (0.1–0.1) | 0/1 (0) |
| Nephritis and renal function | 0 | NA | 0 |
| **Endocrine IMAE** |  |  |  |
| Adrenal insufficiency | 2/8 (25) | 3.9 (0.4–7.3) | 0/8 (0) |
| Hypothyroidism/thyroiditis | 0/8 (0) | NA | 0/8 (0) |
| Hypophysitis | 4/8 (50) | 1.5 (0.1–6.1) | 0/8 (0) |
| Hyperthyroidism | 0/7 (0) | NA | 0/7 (0) |
| Diabetes mellitus | 0/4 (0) | NA | 0/4 (0) |

^a^Includes adverse events considered as potential immune-mediated events by investigator occurring within 100 days of last dose of study drug regardless of causality and treated with immune-modulating medication, except for endocrine events (adrenal insufficiency, hypophysitis, hypothyroidism/thyroiditis, hyperthyroidism, and diabetes mellitus), which were included in the analysis regardless of treatment since these events are often managed without immunosuppression. ^b^Data are reported as number of patients receiving indicated treatment / number of patients who had an IMAE (%).

*NA* not applicable; *IMAE* immune-mediated adverse event; *IMM* immune-modulating medication; *PD-L1* programmed death ligand 1

**Supplementary Fig. 1** CONSORT diagram of patient disposition in the CheckMate 227 study.

**
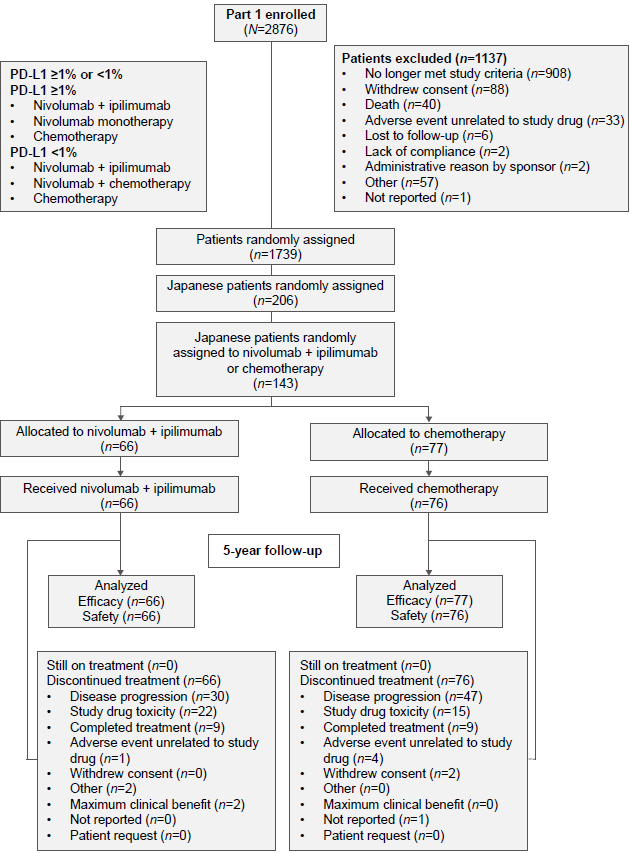
**

**Supplementary Fig. 2** PFS after next line of therapy (PFS2) in Japanese patients with (**a**) tumor PD-L1 expression ≥ 1% and (**b**) tumor PD-L1 expression < 1%. ^a^95% CIs, 24%–53% (nivolumab plus ipilimumab) and 10%–31% (chemotherapy). ^b^95% CIs, 5%–32% (nivolumab plus ipilimumab) and 0–16% (chemotherapy). *CI* confidence interval; *HR* hazard ratio; *NR* not reached; *PD-L1* programmed death ligand 1; *PFS* progression-free survival.

**
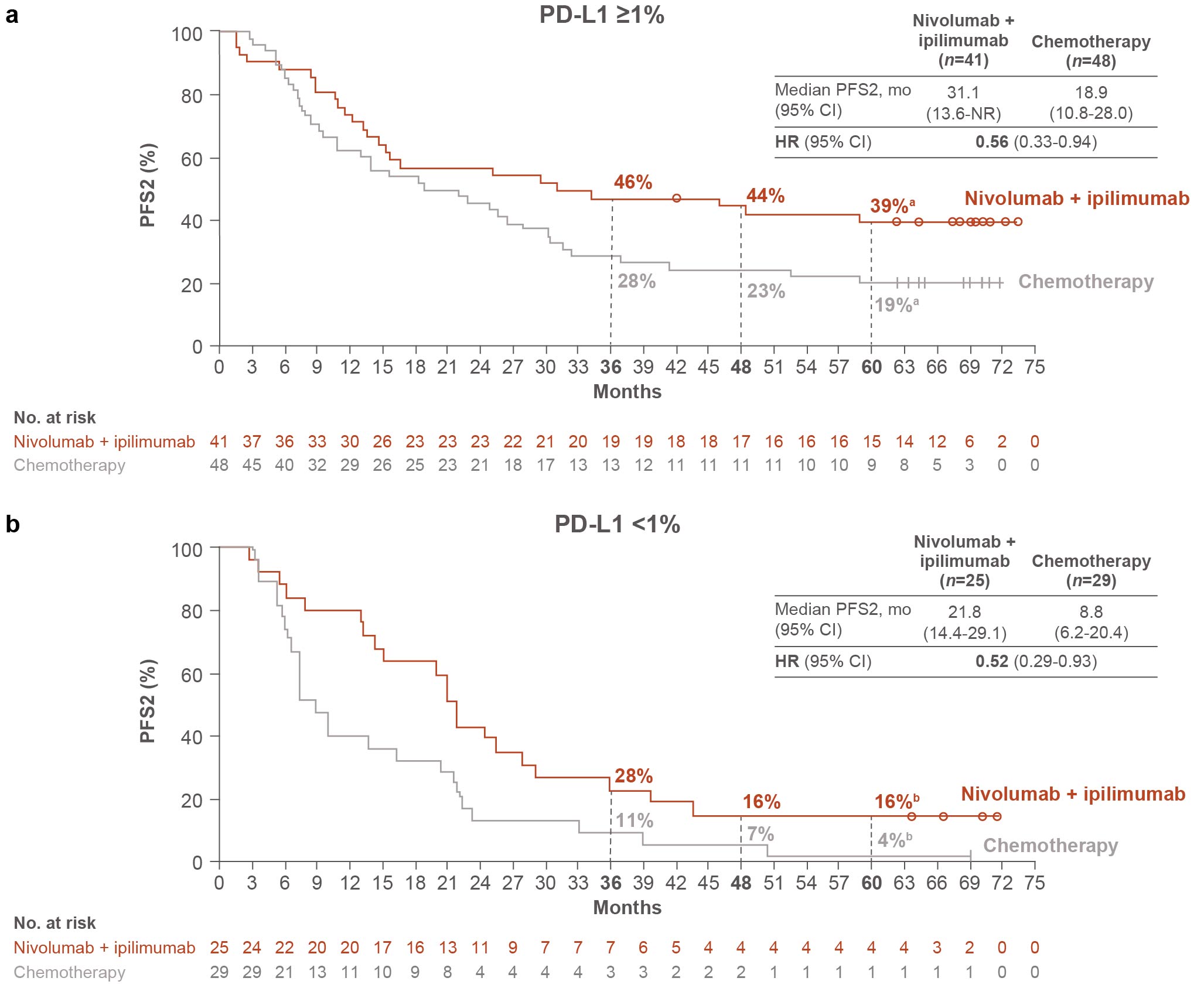
**

**Supplementary Fig. 3** Time to onset (**a**, **b**) and time to resolution (**c**, **d**) of IMAEs in Japanese patients treated with nivolumab plus ipilimumab (PD-L1 ≥ 1% and < 1%). IMAEs included adverse events considered as potential immune-mediated events by investigator occurring within 100 days of last dose of study drug regardless of causality and treated with immune-modulating medication, except for endocrine events (adrenal insufficiency, hypophysitis, hypothyroidism/thyroiditis, hyperthyroidism, and diabetes mellitus), which were included in the analysis regardless of treatment since these events are often managed without immunosuppression. *IMAE* immune-mediated adverse event; *PD-L1* programmed death ligand 1.


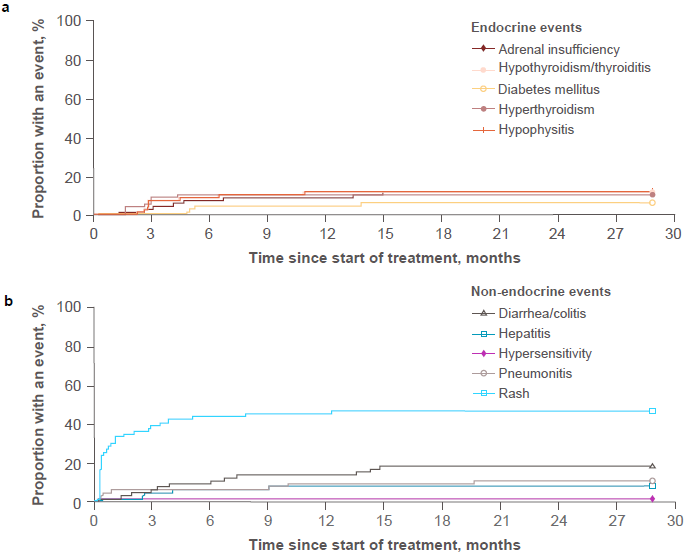


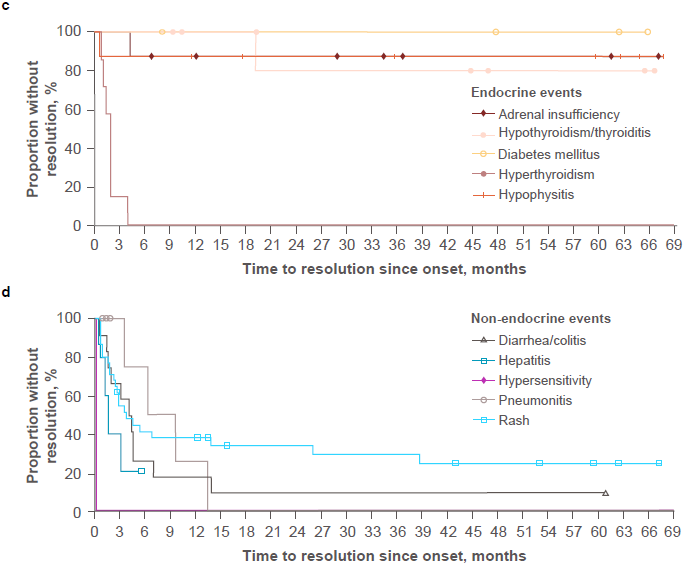

Supplement: Supplementary file 1 — Supplementary file1 (DOCX 543 KB) [file 10147_2023_2390_MOESM1_ESM.docx]
